# Evaluating the efficacy of multiple myeloma cell lines as models for patient tumors via transcriptomic correlation analysis

**DOI:** 10.1038/s41375-020-0785-1

**Published:** 2020-03-02

**Authors:** Vishesh Sarin, Katharine Yu, Ian D. Ferguson, Olivia Gugliemini, Matthew A. Nix, Byron Hann, Marina Sirota, Arun P. Wiita

**Affiliations:** 1 Department of Laboratory Medicine, University of California, San Francisco, CA USA; 2 Bakar Computational Health Sciences Institute, University of California, San Francisco, CA USA; 3 Helen Diller Family Comprehensive Cancer Center, University of California, San Francisco, CA USA; 4 Department of Pediatrics, University of California, San Francisco, CA USA

**Keywords:** Cancer models, Cancer genomics

## Abstract

Multiple myeloma (MM) cell lines are routinely used to model the disease. However, a long-standing question is how well these cell lines truly represent tumor cells in patients. Here, we employ a recently described method of transcriptional correlation profiling to compare similarity of 66 MM cell lines to 779 newly diagnosed MM patient tumors. We found that individual MM lines differ significantly with respect to patient tumor representation, with median *R* ranging from 0.35 to 0.54. ANBL-6 was the “best” line, markedly exceeding all others (*p* < 2.2e−16). Notably, some widely used cell lines (RPMI-8226, U-266) scored poorly in our patient similarity ranking (48 and 52 of 66, respectively). Lines cultured with interleukin-6 showed significantly improved correlations with patient tumor (*p* = 9.5e−4). When common MM genomic features were matched between cell lines and patients, only *t*(4;14) and *t*(14;16) led to increased transcriptional correlation. To demonstrate the utility of our top-ranked line for preclinical studies, we showed that intravenously implanted ANBL-6 proliferates in hematopoietic organs in immunocompromised mice. Overall, our large-scale quantitative correlation analysis, utilizing emerging datasets, provides a resource informing the MM community of cell lines that may be most reliable for modeling patient disease while also elucidating biological differences between cell lines and tumors.

## Introduction

The past 20 years have seen remarkable advances in multiple myeloma (MM) biology and therapy. Many of these discoveries originated with studies performed in MM cell lines. However, there are long-standing questions about the reliability of MM cell lines as models for true disease in patients. One major issue is that while the large majority of MM tumor cells reside within the bone marrow niche, essentially all MM cell lines have been derived from disease growing outside the bone marrow [1]. These patient cells of origin were either circulating in the bloodstream in the form of plasma cell leukemia or in effusions at other sites [2]. In both cases, these cells are expected to have lost reliance on the bone marrow microenvironment for proliferation. Intimate dependence on the marrow niche is a well-known hallmark of typical MM biology [3–5]. However, attempts to establish a long-term culture of MM plasma cells isolated from purely marrow-localized disease have been largely unsuccessful [2]. Therefore, there is ample reason to presume that MM cell lines will carry different phenotypes from patient disease in vivo.

Despite these caveats, cell lines remain the workhorse of MM research. To mitigate these limitations, several groups have developed cell lines that remain dependent on interleukin-6 (IL-6) in culture media [1, 2, 6]. IL-6 is recognized as the most critical bone marrow microenvironment factor supporting MM tumor growth [7–9]. Therefore, these lines may recapitulate additional in vivo phenotypes lost in IL-6 independent lines. In parallel, many attempts have been made to match detected genomic alterations in cell lines, such as translocations or mutations, to specific experimental phenotypes, and then extrapolate these findings to patients with the same genomic lesions (e.g., refs. [10–12]). However, it is unclear how generally the genotype-associated observations in cell lines truly relate to the effects of those genotypes in patient tumor.

Taken together, significant questions remain about both the qualitative and quantitative differences between MM cell lines and patient tumors. Furthermore, it remains unclear whether specific cell lines are more representative of patient tumors than others. Here, we aim to address these questions. Our work extends from our recent study [13], where we correlated RNA-seq data from 666 cell lines in the Cancer Cell Line Encyclopedia (CCLE) to each patient’s RNA-seq data in The Cancer Genome Atlas (TCGA), derived from 8282 tumors, across 22 matching tumor types. The central hypothesis of this approach is that global gene expression patterns provide the most robust phenotypic representation of cellular biology. We specifically identified cell lines that showed greatly increased and decreased global transcriptomic correlations versus primary patient samples. Based on these results, we proposed that the cell lines used in the standard “NCI-60” preclinical panel should be replaced by a “TCGA-110-CL,” employing a cohort of lines with the most similarity to patient tumors. In parallel, others have also used transcriptional correlation profiling to suggest the best cell line models of metastatic breast cancer [14] and hepatocellular carcinoma [15], for example, demonstrating the widespread utility of this approach.

As the TCGA primarily includes data on solid tumors, our prior publication did not include MM. Fortunately, the Multiple Myeloma Research Foundation (MMRF) has addressed this gap in knowledge. The MMRF has sponsored a comprehensive transcriptomic resource of MM cell lines (www.keatslab.org) and MM patient tumors within the MMRF CoMMpass study (research.themmrf.org). Here, we employed our transcriptional correlation profiling approach to perform 51,414 individual correlations of cell lines versus patient tumor. We confirmed that MM cell lines and patient tumors display broad transcriptomic differences. However, we did identify cell lines, in particular ANBL-6, that appear to be more representative of patient disease than others. In contrast, some widely used lines scored relatively poorly in our ranking of similarity to patients. We further characterized additional features to aid in increasing similarity of cell lines to patient tumor. Here, we provide a resource for cell line selection in MM research while also elucidating underlying biological signatures distinguishing cell lines and patient tumors.

## Materials and methods

### Transcriptome and mutational analysis

See details of analysis in Supplementary Methods. Briefly, annotated read count data were obtained from Keats lab cell line (www.keatslab.org) and CoMMpass IA13 patient datasets (research.themmrf.org) and normalized via variance stabilizing transformation [16]. The top 5000 most variable genes were used for Spearman correlation analyses. Exome sequencing-based mutation data were similarly obtained from annotated datasets from these resources. Clinical subset analysis was performed as annotated for patients in CoMMpass. CoMMpass patient translocations were annotated as in ref. [17].

### ANBL-6 experiments

See details of analysis in Supplementary Methods. Briefly, ANBL-6 cell lines were stably transduced with a lentiviral construct that stably expressed enhanced firefly luciferase and was implanted into female 6–8 week old NOD *scid* gamma (NSG) mice. Tumor burden was monitored by bioluminescent imaging.

## Results

### Global correlation analysis reveals that MM cell lines are not equal representations of patient tumors

We began by obtaining RNA-seq read count data for 66 MM cell lines (Keats lab resource) and CD138+ enriched tumor cells from 779 newly diagnosed MM patients (CoMMpass release IA13). As in our prior study [13], we normalized all reads using the upper-quartile method via Variance Stabilizing Transformation [16] (see “Materials and methods”). We note that all cell line and patient RNA sequencing libraries were prepared and analyzed in the same laboratory (Jonathan Keats lab at TGEN), reducing potential for artifacts when comparing samples generated from different groups.

**Fig. 1:**
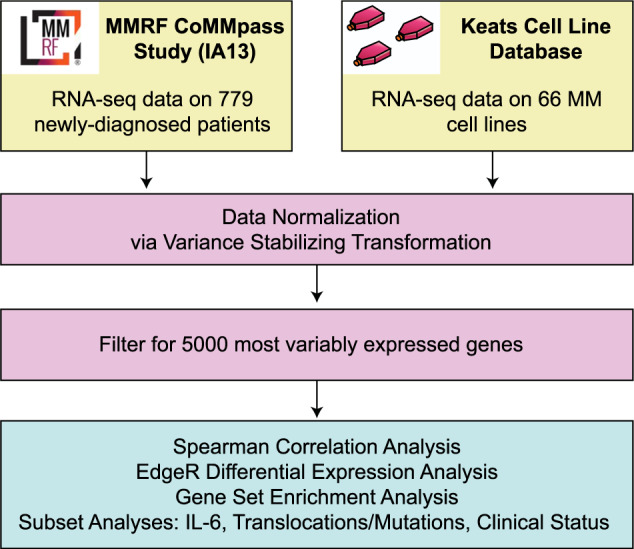
Workflow for RNA-seq based correlation analysis of multiple myeloma cell lines and patients. The workflow describes the datasets used as well as analysis strategies.

**Fig. 2:**
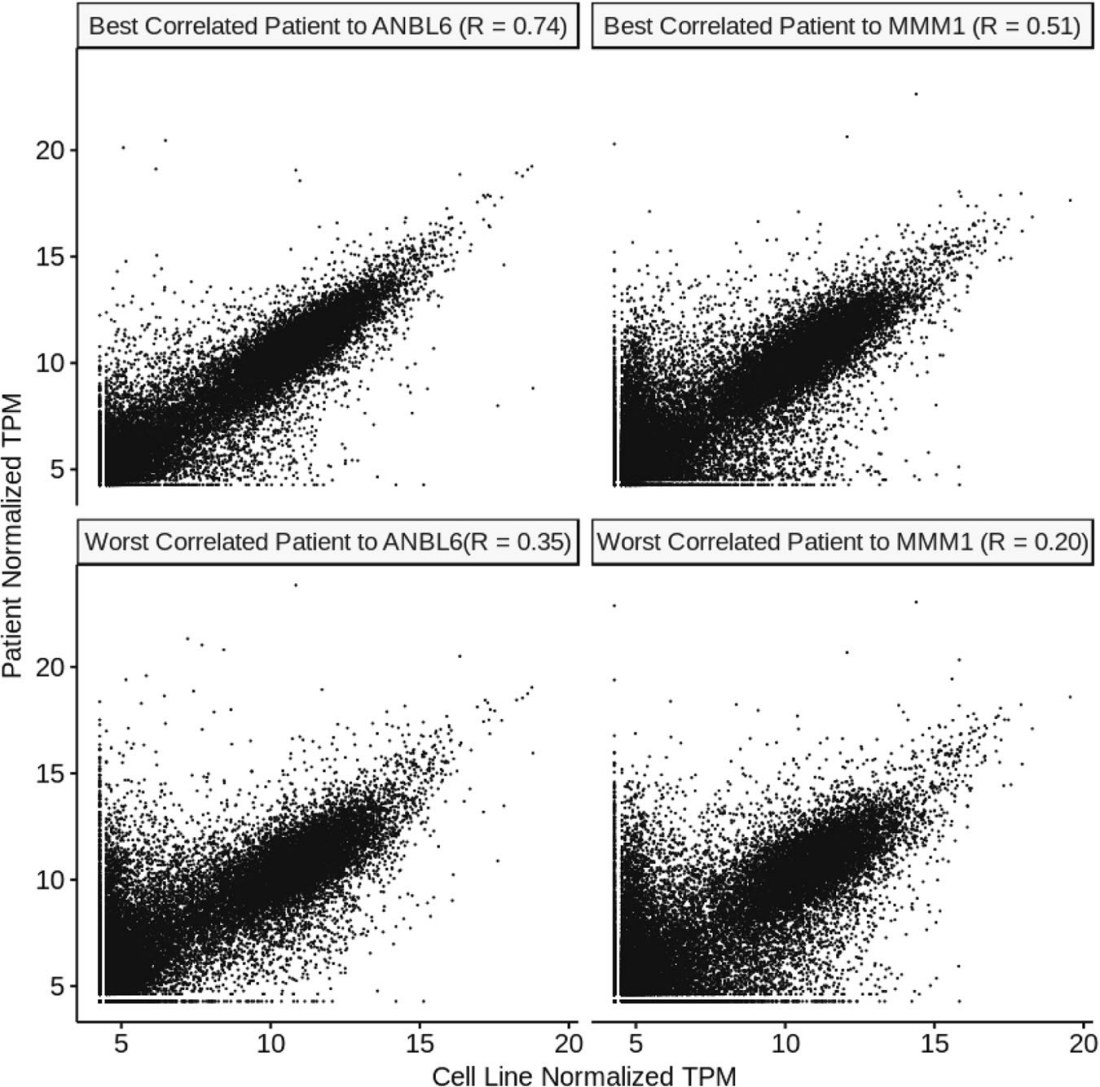
Example correlation plots for individual patients and cell lines. We show here examples of the best cell line (ANBL-6) and the worst cell line (MMM1) from our ranking in Fig. 3. Gene expression in transcripts per million (TPM) from RNA-seq data is plotted versus gene expression for the highest correlating and lowest correlating patient for each cell line. Similar correlations underpin the other analyses performed throughout this work.

**Fig. 3:**
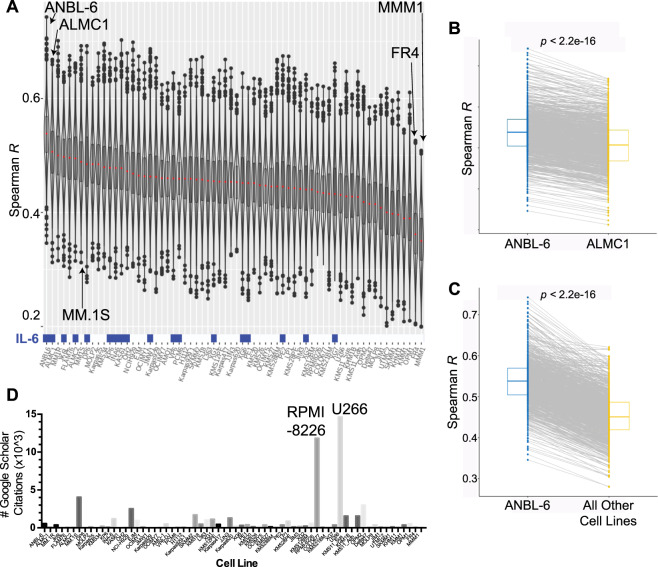
Overall MM cell line rankings reveal more and less patient-like in vitro disease models. **a** Correlation analysis of the CCLE and CoMMpass data. Each sample in the violin plot corresponds to the Spearman correlation between one cell line and one primary tumor sample using the 5000 most variable genes. In the overlaid box plot, the red center line depicts the median, the box limits depict the upper and lower quartiles, and the whiskers depict 1.5 times the interquartile range. Culture with IL-6 prior to RNA-seq analysis is indicated as blue boxes at bottom of plot. **b** Comparison of each patient’s Spearman correlation to ANBL-6 versus the second-ranked line, ALMC-1, demonstrates the highly significant increased correlation with ANBL-6. **c** Similarly, essentially all patient transcriptomes correlate more strongly with ANBL-6 transcriptome than the aggregate panel of all other cell lines. All *p* values using Wilcoxon test. **d** The literature usage for each cell line was measured using a Google Scholar search (October 2, 2019). The number of individual results from the text search of “[cell line] myeloma” is plotted per cell line and ordered per the rankings in 3a.

As in our prior study, we focused our analysis on the top 5000 most variable genes across samples expressed consistently at >1 counts per million, with the reasoning that these genes are most likely to be biologically informative for similarity assessment (see Supplementary Methods). A workflow for our analysis is shown in Fig. 1. Our primary analysis is performing a Spearman correlation across these 5000 genes for each cell line versus each patient tumor sample, with the hypothesis that a perfect correlation (*R* = 1) means that a cell line is an exact representation of the patient tumor. We show individual correlation plots in Fig. 2 to provide examples of the 51,414 total correlations performed to generate our overall rankings in Fig. 3a.

The violin plots in Fig. 3a are presented for each cell line in the Keats lab database, ranked by the median Spearman *R* when correlated versus each patient in the CoMMpass database. We can draw some initial conclusions from this dataset. First, it is clear that none of the MM cell lines approached a perfect representation of patient tumor, as the median *R* values range from 0.35 to 0.54 (i.e., far from 1). Consistent with this conclusion, principal component analysis of overall transcript expression demonstrated that MM cell lines form a distinct cluster from patient tumors (Fig. S1). Second, while many of the cell lines in the middle of the ranking showed quite similar correlations to patient tumor, the cell line ANBL-6 sat atop the ranking as a notable outlier (median *R* = 0.54). In parallel, the cell lines MMM.1 and FR4 appeared markedly below other lines (median *R* = 0.35 and 0.36, respectively).

We further evaluated these findings by comparing the paired analysis of each patient tumor correlated with ANBL-6 versus the second-ranked cell line, ALMC-1 (Fig. 3b), as well as all other cell lines (Fig. 3c). In both cases, ANBL-6 led to significantly higher correlations (Wilcoxon *p* < 2.2e−16). Similarly, MMM.1 and FR4 led to significantly worse correlations versus all other lines (Fig. S2).

These results support the notion that while no MM cell line is perfect, some are still better (or worse) than others. Notably, we compared our cell line rankings to the frequency of use of MM cell lines in the literature (Google Scholar, October 2, 2019) (Fig. 3d). As expected, the well-known and earliest-established [2] lines RPMI-8226 and U-266 emerged at the top of the citation ranking. However, a quick glance revealed that these lines are not localized to the top of the patient similarity rankings. U-266, for example, despite its number 1 citation rank at ~14,600 mentions in the literature, actually appeared to be one of the lesser-representative lines (rank 52 of 66). RPMI-8226, with 11,800 uses in the literature, ranked 48. Our top cell line, ANBL-6, is certainly used in the literature, with 563 publications employing it, but still only comes in at number 16 in the citation rankings (Dataset S1). Fortunately, MMM.1 and FR4 are only rarely used in the literature (56 and 64 uses, respectively). Overall, these results indicate that frequency of appearance in the literature does not strongly predict whether a cell line actually well represents patient tumor.

### Cell line rankings are largely consistent across laboratories

Decades of anecdotal experience have suggested that cell lines may demonstrate phenotypic “drift” when cultured in different laboratories. Recent large-scale, multiomic studies have systematically confirmed and quantified these effects [18, 19]. Therefore, we used an orthogonal resource, the CCLE [20], to evaluate whether our rankings still hold when based on RNA-seq data generated by an entirely different group. Fortunately, we were able to examine a substantial cohort of 25 overlapping cell lines between the CCLE and Keats databases (Fig. S3A).

**Fig. 4:**
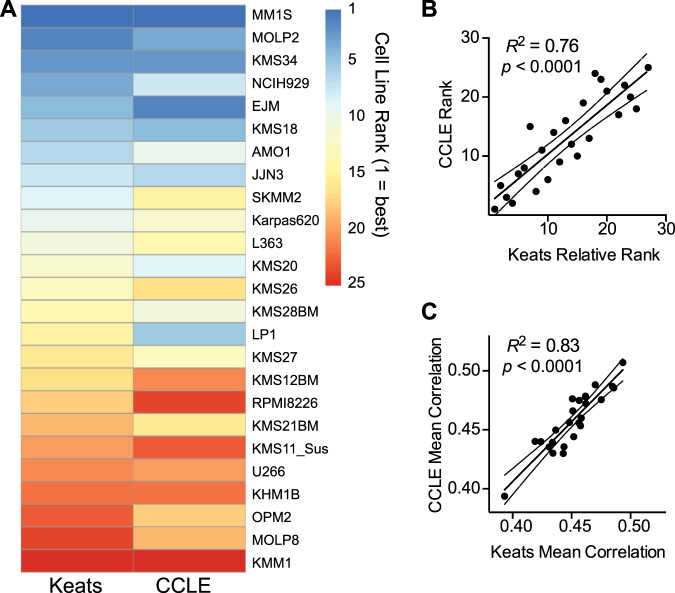
Cell line correlation rankings are largely reproducible across RNA-seq datasets from different laboratories. **a** We performed our correlation rankings using RNA-seq data from 25 MM cell lines available in both the CCLE and Keats lab databases, with each line compared with all patients in CoMMpass. Cell lines at the top of the ranking (blue) tend to remain at the top in both rankings, and those at the bottom (red) tend to remain at the bottom in both rankings. **b** Further supporting reproducibility, numerical rankings in CCLE and relative rankings in Keats database (numbered 1–25 to reflect rank order in overall 66 cell line ranking) are highly correlated. **c** Similarly, mean Spearman *R* of transcriptome between each cell line and all patients, as determined from each database, is highly consistent. Linear regression displayed with 95% confidence intervals.

We were encouraged to find strong consistency between the rankings generated from both cell line datasets (Fig. 4a). Upon visual inspection it is clear that the same cell lines show a notable tendency to stay near the top and bottom of both rankings. Quantitative comparison of rankings and median Spearman correlations also demonstrated high reproducibility (Fig. 4b, c). Furthermore, statistical analysis also confirmed that there is no significant difference between the median correlations generated from each cell line dataset (Fig. S3B). Notably, both rankings assert that the most commonly used lines RPMI-8226 and U-266 are relatively poor representatives of patient tumor. In contrast, among frequently used lines, MM.1S retains one of the highest scores by both rankings. While CCLE does not include ANBL-6, the reproducibility of the overall ranking increases confidence that this top-ranked line in the Keats dataset will also show similar patient-representative gene expression patterns when used in other laboratories.

### Culture with IL-6 drives similarity between cell line and patient tumor transcriptome

**Fig. 5:**
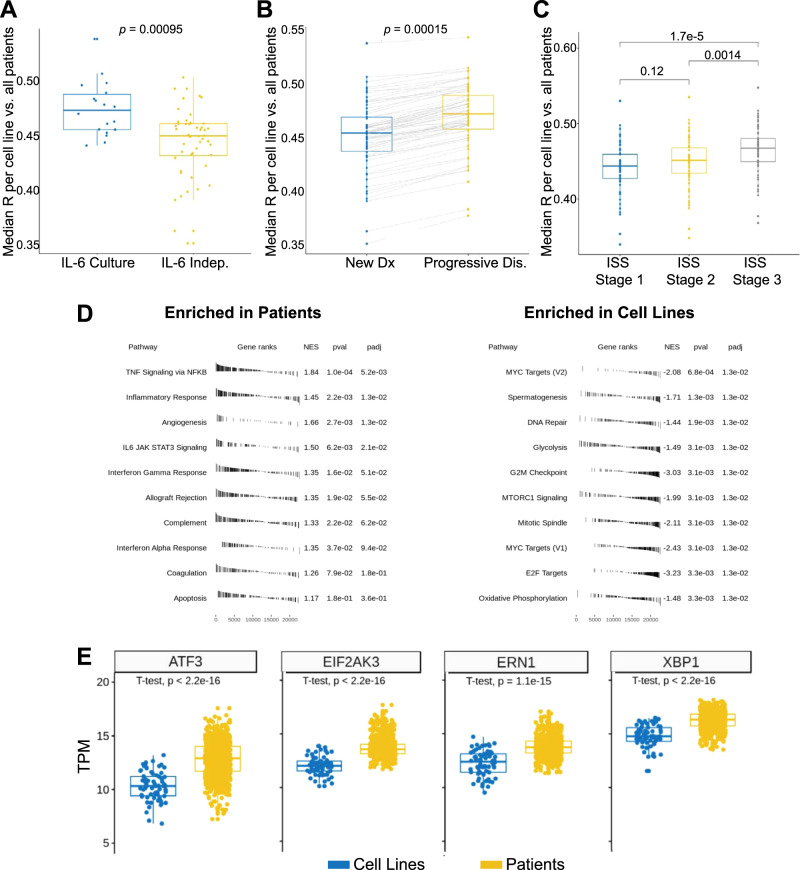
Biological factors drive increased correlations and overall differences between patient tumors and cell lines. **a** Box plots (each dot median of a cell line compared with all patients in CoMMpass) indicate that culture with IL-6 significantly increases the similarity of cell lines to tumors. **b** The cohort of CoMMpass patients with progressive disease showed increased similarity to cell lines versus newly diagnosed. **c** Increased international staging system (ISS) grade at diagnosis leads to more similarity to cell lines. **d** Gene Set Enrichment Analysis (GSEA) reveals immune signaling signatures significantly enriched in patient tumors, whereas signatures of proliferation and oncogenesis are enriched in cell lines. Performed using cutoff of differentially expressed genes at Log2 fold-change > |1|, false discovery rate < 0.01. **e** Comparative expression of selected genes (in transcripts per million, TPM) related to unfolded protein stress identifies differences in cell lines and patients. *p* values by Wilcoxon test in 5a-5c; by two-sided *t*-test in 5e.

We noted that ANBL-6, our top-ranked line, was initially characterized as being dependent on IL-6 [21]. We therefore tested the hypothesis that culture of cell lines with IL-6 generally produces a more “patient-like” transcriptional signature. Indeed, we found this to be the case, where lines cultured in IL-6 in the Keats dataset showed a significant improvement in median correlation versus all patient tumors (Wilcoxon *p* = 9.5e−4) (Fig. 5a). While the absolute difference in the correlations across cell lines is modest (mean *R* = 0.48 for IL-6 cultured versus 0.45 without IL-6), we do note that lines cultured in IL-6 show a clear enrichment in the top half of the rankings (Fig. 3a). Furthermore, while the annotated lines were cultured with IL-6 for RNA-seq analysis, many were subsequently found to not actually be dependent on this cytokine (https://www.keatslab.org/projects/mm-cell-line-characterization/cell-line-characterization-status). This finding suggests that coculture with critical microenvironment factors can at least partially drive cell lines to a more patient-like phenotype, even if not strictly required for cell growth.

### Poor-prognosis clinical features drive similarity between cell lines and patient tumors

We next surmised that MM plasma cells able to grow in vitro are likely selected for increased proliferative (PR) capacity. As a corollary, patients with more aggressive disease may therefore have tumors with more similarity to cell lines. To test this, we first analyzed newly diagnosed patients in CoMMpass based on international staging system (ISS) stage at diagnosis (Fig. 5b). We indeed found that the poorest-prognosis patients, at ISS stage 3, had tumors with transcriptional profiles significantly more similar to all cell lines together (*p* = 0.0014 and 1.7e−5 versus stage 2 or stage 1, respectively). Similarly, patients with higher M spike at baseline also showed greater tumor similarity to cell lines (Fig. S4).

We also evaluated the smaller cohort of patients with progressive disease in CoMMpass (*n* = 81) and obtained their overall transcriptional correlations to all cell lines, in comparison with our prior newly diagnosed analysis (*n* = 779). Here we also found significantly increased correlation with the relapsed versus newly diagnosed patients (*p* = 0.0015) (Fig. 5c). Taken together, our results provide quantitative support for the notion that MM cell lines more closely resemble more aggressive, poor-prognosis disease states rather than the “typical” newly diagnosed myeloma patient.

### Specific biological signatures differentiate cell lines and patient tumors

While these results help clarify the basis of MM cell line phenotypes, we next used Gene Set Enrichment Analysis to further delineate biological signatures that mostly distinguish cell lines and patient tumors (Fig. 5d). We found that signatures relating to cell cycle/proliferation, mTOR signaling, and MYC targeting were significantly upregulated in cell lines versus patient samples. These findings underscore how MM cell lines have adapted to the setting of rapid, cell-autonomous proliferation in vitro. In contrast, patient tumors showed increased signatures of immune and microenvironment signaling including IL-6/JAK/STAT3 signaling, interferon response, TNF signaling, and complement. Similar findings were obtained with Gene Ontology analysis (Fig. S5). These results illustrate the importance of MM immune microenvironment effects in driving human in vivo transcriptional phenotypes. These results also suggest that exposing cell lines to more of these microenvironmental factors, including, but not limited to, IL-6, may assist in further driving a patient-like signature in vitro.

Furthermore, it has been reported that MM cell lines frequently downregulate immunoglobulin synthesis compared with patient tumors [22, 23]. We also observed this phenomenon comparing expression of all transcripts of patients versus cell lines (Fig. S6 and Dataset S2). We hypothesized this decreased protein load would lead to a decrease in baseline unfolded protein stress in cell lines. Indeed, we also found decreased cell line expression of genes that govern protein homeostasis and unfolded protein stress in the endoplasmic reticulum, such as ATF3, EIF2AK3/PERK, XBP1, and ERN1/IRE1, when compared with patients (Fig. 5e). Given the prominent role of misfolded immunoglobulin burden in proteasome inhibitor-induced apoptosis [24–26], this result may partially explain why MM cell lines are not markedly more sensitive to bortezomib than many solid tumor cell lines, whereas among cancer patients only those with MM have shown strong clinical responses to proteasome inhibition [27].

### MM cell lines show increased *TP53* mutation frequency compared with patient tumors

Over the past 10 years, large-scale whole genome and whole exome sequencing studies have revealed numerous mutations found recurrently in MM [28–30]. These findings follow prior cytogenetic studies which have found large-scale chromosomal aberrations, including both translocations and copy-number variants, that drive differential patient prognosis and are routinely tested in the clinical setting [31].

Here we took advantage of whole exome sequencing data in CoMMpass and the Keats lab cell line database to investigate the relative frequency of mutations in both sample sets (Fig. S7). We first note that activating mutations in the most recurrently altered oncogenes in patients, *KRAS* and *NRAS*, are mutated at similar frequency in both cell lines and patient samples (30.9% versus 25.1% *KRAS*, 20.1% versus 21.1% *NRAS*, respectively) demonstrating consistency between these key sequence variants. *TP53* mutations were markedly more common in cell lines (55.9% versus 4.1% in patients), potentially consistent with the more aggressive growth phenotype of cells in vitro. Other commonly mutated genes in patient tumors, as characterized by Walker et al. [30], generally show similar mutation frequencies in cell lines and patients. Beyond these well-known genes, we did not identify significantly different mutational profiles in any genes consistently expressed at the mRNA level in both cell lines and patients (not shown).

### Matching of common MM genomic aberrations does not always lead to increased cell line-patient transcriptional similarity

**Fig. 6:**
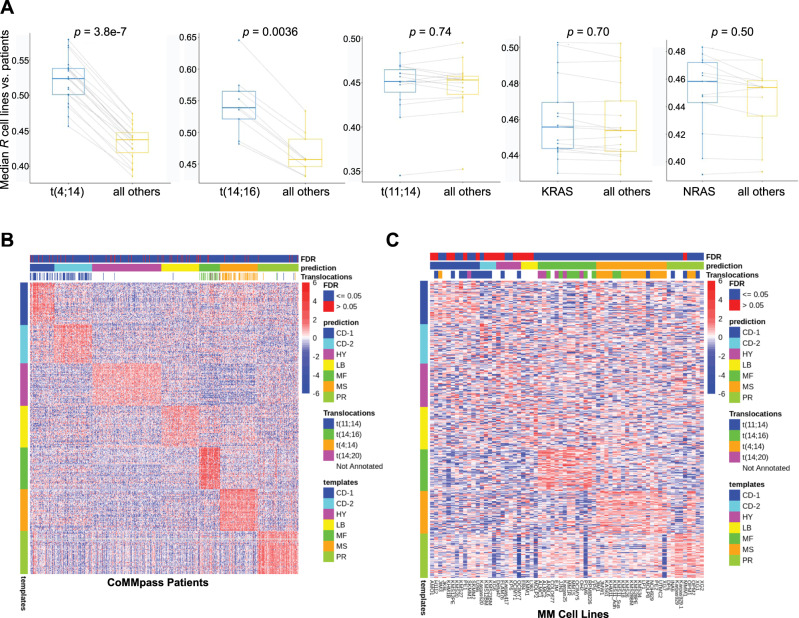
Integrated genomic and transcriptomic analysis to compare patient tumor versus cell lines. **a** Subset analysis of correlation profiling when matching canonical myeloma genomic lesions (three *IGH* translocations, *KRAS*/*NRAS* mutations). Each dot reflects the median Spearman correlation of each cell line carrying the specified genomic lesion correlated versus CoMMpass patients with or without the noted genomic lesion. Box plot shows median and interquartile range. *p* values by Wilcoxon test. **b** Heatmap showing patient expression levels of canonical genes (*y*-axis) overexpressed in each of the seven myeloma subtypes per the analysis of Zhan et al. (“template” = subtype labels as defined by Zhan et al.). Each CoMMpass patient transcriptome was classified into one of the myeloma subtypes based on their relative expression of these canonical genes using the Nearest Template Prediction method (see Methods). False discovery rate (FDR) < 0.05 denotes high-confidence match. Translocations annotated in each sample are also noted and demonstrate close matches to the expected subtypes defined primarily by a chromosomal alteration in Zhan et al. **c** Similar analysis as in **b** but for 66 MM cell lines. In this analysis, differential gene expression driving a specific subtype match appears less prominent than in patients. No strong matches are noted for cell line transcriptomes to the HY, LB, and CD-2 subtypes. For **b** and **c**, Heatmap scale bar reflects log2-normalized gene expression data with 0 as the median in each row.

In MM research it is common to use cell lines with particular genomic lesions as proxies for biological features for patients with the same aberrations. We next tested whether some of these most-common genomic aberrations—translocations (11;14), (4;14), and (14;16), as well activating mutations of *NRAS* and *KRAS* (codons 12/13/61)—improved global transcriptomic correlations when matched between cell lines with patients carrying the same lesion. Our analysis confirmed that matching of *t*(4;14) and *t*(14;16) cell lines indeed improved correlations to patients with the same alteration as compared with those without (*p* = 3.8e−7 and 0.0036, respectively) (Fig. 6a, left). However, we saw no significant increase when matching *t*(11;14) or activating *RAS* mutations (Fig. 6a, right). While these results by no means refute the utility of extrapolating findings from cell lines with specific aberrations to patients with the same genotype, they do surprisingly indicate that these latter genotypes do not lead to broad-scale increases in the global cellular transcriptome similarity based on the presence of the same lesion.

### Clustering analysis reveals many cell lines do not align with canonical patient subtypes

Microarray-based transcriptome profiling by Zhan et al. [32] previously demonstrated that myeloma patient tumors can be divided into seven molecular subtypes. We clustered all CoMMpass patient tumors based on the characteristic gene signatures of each Zhan et al. subtype, confirming that CoMMpass samples well-align across the reported groups (Fig. 6a). However, we found markedly different results for cell lines (Fig. 6b). Consistent with our findings above, we found the strongest alignment to genes defining the “MF” and “MS” subtypes, characterized by *t*(14:16)/*t*(14;20) and *t*(4;14), respectively. As indicated by several matches with a false discovery rate >0.05, we found a weaker match of any cell lines to the CD-1 subtype, characterized by *t*(11;14) disease. We also noted several cell lines best matching to the “proliferative” subtype. However, in contrast to patient tumors, almost all cell lines showed at least moderate expression of the PR signature, potentially consistent with the ability to propagate rapidly in vitro. Finally, we noted that none of the cell lines showed strong matches to the “low bone” (LB), CD-2, and hyperdiploid (HP) gene expression signatures. The latter finding particularly emphasizes that no MM lines well recapitulate the common HP genotype. This result underscores a major difference between available in vitro MM models and in vivo disease, while also suggesting that HP disease may be highly dependent on the tumor microenvironment.

We further assessed whether unbiased hierarchical clustering of MM cell line transcriptomes across the 5000 genes used in Fig. 3a may reveal alternate subtypes among cell lines only (Fig. S8). In this analysis we identified clustering based on *t*(4;14) and *t*(14;16)/*t*(14;20) signatures, similar to that noted above, but did not identify other prominent drivers of similarity across the landscape. We did note, however, that ANBL-6 clustered closely with other highly ranking cell lines MM.1S, MM.1R, ALMC-1, and ALMC-2, suggesting that these lines may carry a common transcriptional signature driving similarity to patient tumor.

### ANBL-6 is appropriate for disseminated in vivo MM modeling

Our overall rankings (Fig. 3a) suggest that ANBL-6 should be incorporated more frequently into MM studies. Toward more widespread use of ANBL-6, one potential drawback for MM in vitro studies is the cost of recombinant IL-6. We therefore titrated IL-6 and found that over 72 h, a minimal concentration of 0.1 ng/mL was able to support equivalent proliferation to 100 ng/mL (Fig. S9), consistent with earlier results [33]. No proliferation was observed in the absence of IL-6, confirming IL-6 dependence.

Furthermore, while ANBL-6 has been used in many prior studies (Fig. 2d), the vast majority of the efforts were purely in vitro. Disseminated orthotopic xenograft models of MM, where luciferase-labeled plasma cells are intravenously (IV) implanted in NOD *scid* gamma (NSG) mice, may carry significant advantages for preclinical modeling if tumor cells home to hematopoietic tissues including bone marrow [34]. In this context, cells will proliferate and respond to therapy in a microenvironment more akin to that present in patients.

**Fig. 7:**
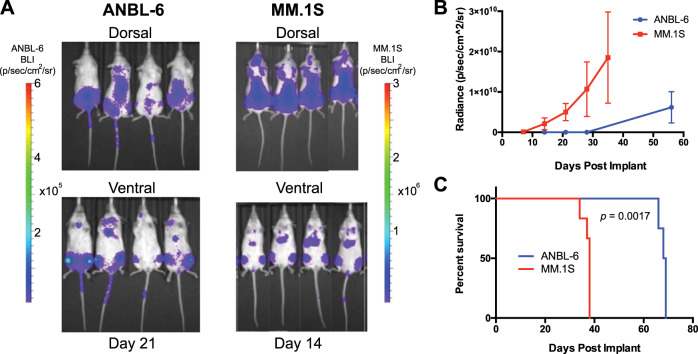
Investigating the potential of ANBL-6 for preclinical in vivo modeling in myeloma. **a** 1e6 luciferase-labeled ANBL-6 and MM.1S cells were implanted via tail vein injection into NSG on the same date. Bioluminescence imaging (BLI) data are shown at noted dates post implant. Both cell lines show identical localization, primarily to long bones of hindlimbs and to the spine. Note scale bar with lower intensities for BLI signal in ANBL-6. **b** Quantification of BLI signal (*n* = 4 in ANBL-6 arm; *n* = 6 in MM.1S arm). **c** Survival of MM.1S-implanted mice versus ANBL-6 implanted mice illustrates more indolent course of ANBL-6 in vivo. *p* value by log-rank test.

To our knowledge, it has not been tested whether ANBL-6 homes to hematopoietic tissues in a disseminated mouse model after IV implant. We therefore used lentiviral transduction to stably express luciferase in ANBL-6 cells and injected 1e6 cells into a pilot cohort of four NSG mice. In parallel, we injected 1e6 luciferase-labeled MM.1S cells into a separate cohort as a control, as these cells are well known to home to bone marrow in NSG mice [35]. Encouragingly, we found that ANBL-6 showed an identical pattern of distribution as MM.1S, with implantation primarily to the spine, sternum, and hindlimbs (Fig. 7a). However, in vivo growth kinetics and overall murine survival were significantly prolonged compared with MM.1S (Fig. 7b, c). Therefore, ANBL-6 may perhaps serve as a valuable in vivo model for a more indolent form of MM, rather than highly aggressive disease as represented by most disseminated MM cell line models.

## Discussion

Here we present the first large-scale quantitative comparison of MM cell lines and primary patient tumors. Our results, using global transcriptome profiles as a proxy for overall biological state, quantitatively confirm long-standing suspicions that MM cell lines are indeed very different than patient tumors. Through these analyses, we describe biological factors that drive increased or decreased similarity between cell lines and patients, as well as outline strategies to potentially improve the quality of in vitro studies toward representing in vivo patient disease.

One potential explanation for the overall low correlations between cell lines and patient transcriptomes is that cell lines are typically derived from multiply relapsed, extra-medullary samples that have lost dependency on the bone marrow microenvironment. This biological setting is a significant contrast to the newly diagnosed, marrow-based disease included in CoMMpass. Against this background, though, we argue that it is still certainly possible to improve the quality and relevance of in vitro studies by incorporating our rankings and other findings here. Even though many of our comparative analyses (IL-6 coculture, relationship to progressive disease, etc.) show relatively modest absolute increases in global transcriptional correlation, these increases remain highly statistically significant and involve biologically relevant changes over hundreds of genes.

We note that our analysis here is only enabled by recent, large-scale RNA sequencing-based studies in MM. Prior microarray-based expression profiling analyses of patient samples [36], for example, do not readily allow for similar robust normalization and quantification approaches when comparing across different datasets. In parallel, though, our findings here are of course limited by the fact that they are solely based on the transcriptome. Other “omic” signatures (metabolomics, proteomics, etc.), or, alternatively, curated individual markers, may further refine and extend these results. However, given current technologies, we would argue that transcriptional profiles are the best way to directly assess the relationship of these cell line models to patient disease.

Similar to our prior pan-cancer analysis of the TCGA and CCLE [13], here we identify MM cell line models that appear both more and less representative of patient disease. In particular, our results suggest that the two most widely used MM cell lines in the literature, U-266 and RPMI-8226, are actually some of the least representative of patient disease (Fig. 3). In contrast, we find that the third-most-used cell line, MM.1S, does appear to be one of the better models available. Importantly, these findings were reproducible across two different datasets (Fig. 4).

Our results specifically indicate that the cell line ANBL-6 sits significantly above all other cell lines in terms of patient similarity. ANBL-6 was isolated from peripheral blood of a relapsed MM patient and initially characterized as having typical malignant plasma cell immunophenotype, a (14;16) translocation, and lambda light chain secretion [21], and later shown to have wild-type *NRAS* and *KRAS* sequences [10, 33]. We confirmed that ANBL-6 showed consistent proliferation even at a minimal, cost-effective IL-6 concentration. Notably, our prior results suggest that other factors within the NSG murine marrow microenvironment may be able to partially compensate for the lack of cross talk between murine IL-6 and human IL-6 receptor [35], thereby allowing for in vivo proliferation of ANBL-6. Furthermore, we found that coculture with IL-6 appears to drive more patient-like phenotypes across all cell lines (Fig. 5a), and IL-6-mediated signaling is a prominent transcriptome signature enriched in patient tumors (Fig. 5d). We therefore propose two readily implemented actions based on our results: (1) more widespread use of ANBL-6 with decreased use of RPMI-8226 and U-266; and (2) more common use of IL-6 in culture media, potentially even for lines not strictly dependent on this cytokine.

The overall conclusions here will not necessarily be surprising to MM researchers given the years of anecdotal experience and knowledge of MM biology. However, like recent studies systematically investigating differences in cell line phenotype across different laboratories [18, 19], these results are important to provide quantifiable metrics to compare cell lines and patient tumors, and potentially provide benchmarks for the development of new lines. The analyses here stand as a resource with widespread utility to the MM community and lead to specific recommendations for alterations in research practice.

## Supplementary information

Supplementary Materials

Dataset S2

Dataset S1

## References

[1] Drexler HG, Matsuo Y (2000). Malignant hematopoietic cell lines: in vitro models for the study of multiple myeloma and plasma cell leukemia. Leuk Res.

[2] Jernberg-Wiklund H, Nilsson K. Multiple myeloma cell lines. In: Masters JRW & Palsson BO, editors. Human cell culture Vol. III. Cancer Cell Lines Part 3: Leukemias and Lymphomas. Springer (Netherlands) 2002. p. 81–155.

[3] Kawano Y, Moschetta M, Manier S, Glavey S, Gorgun GT, Roccaro AM (2015). Targeting the bone marrow microenvironment in multiple myeloma. Immunol Rev.

[4] Manier S, Kawano Y, Bianchi G, Roccaro AM, Ghobrial IM (2016). Cell autonomous and microenvironmental regulation of tumor progression in precursor states of multiple myeloma. Curr Opin Hematol.

[5] Kuehl WM, Bergsagel PL (2012). Molecular pathogenesis of multiple myeloma and its premalignant precursor. J Clin Invest.

[6] Hawley RG, Berger LC (1998). Growth control mechanisms in multiple myeloma. Leuk Lymphoma.

[7] Brocke-Heidrich K, Kretzschmar AK, Pfeifer G, Henze C, Loffler D, Koczan D (2004). Interleukin-6-dependent gene expression profiles in multiple myeloma INA-6 cells reveal a Bcl-2 family-independent survival pathway closely associated with Stat3 activation. Blood.

[8] Hideshima T, Nakamura N, Chauhan D, Anderson KC (2001). Biologic sequelae of interleukin-6 induced PI3-K/Akt signaling in multiple myeloma. Oncogene.

[9] Klein B, Zhang XG, Lu ZY, Bataille R (1995). Interleukin-6 in human multiple myeloma. Blood.

[10] Billadeau D, Liu P, Jelinek D, Shah N, LeBien TW, Van Ness B (1997). Activating mutations in the N- and K-ras oncogenes differentially affect the growth properties of the IL-6-dependent myeloma cell line ANBL6. Cancer Res.

[11] Paterson JL, Li Z, Wen XY, Masih-Khan E, Chang H, Pollett JB (2004). Preclinical studies of fibroblast growth factor receptor 3 as a therapeutic target in multiple myeloma. Br J Haematol.

[12] Touzeau C, Dousset C, Le Gouill S, Sampath D, Leverson JD, Souers AJ (2014). The Bcl-2 specific BH3 mimetic ABT-199: a promising targeted therapy for t(11;14) multiple myeloma. Leukemia.

[13] Yu K, Chen B, Aran D, Charalel J, Yau C, Wolf DM (2019). Comprehensive transcriptomic analysis of cell lines as models of primary tumors across 22 tumor types. Nat Commun.

[14] Liu K, Newbury PA, Glicksberg BS, Zeng WZD, Paithankar S, Andrechek ER (2019). Evaluating cell lines as models for metastatic breast cancer through integrative analysis of genomic data. Nat Commun.

[15] Chen B, Sirota M, Fan-Minogue H, Hadley D, Butte AJ (2015). Relating hepatocellular carcinoma tumor samples and cell lines using gene expression data in translational research. BMC Med Genom.

[16] Durbin BP, Hardin JS, Hawkins DM, Rocke DM (2002). A variance-stabilizing transformation for gene-expression microarray data. Bioinformatics.

[17] Barwick BG, Neri P, Bahlis NJ, Nooka AK, Dhodapkar MV, Jaye DL (2019). Multiple myeloma immunoglobulin lambda translocations portend poor prognosis. Nat Commun.

[18] Ben-David U, Siranosian B, Ha G, Tang H, Oren Y, Hinohara K (2018). Genetic and transcriptional evolution alters cancer cell line drug response. Nature.

[19] Liu Y, Mi Y, Mueller T, Kreibich S, Williams EG, Van Drogen A (2019). Multi-omic measurements of heterogeneity in HeLa cells across laboratories. Nat Biotechnol.

[20] Barretina J, Caponigro G, Stransky N, Venkatesan K, Margolin AA, Kim S (2012). The Cancer Cell Line Encyclopedia enables predictive modelling of anticancer drug sensitivity. Nature.

[21] Jelinek DF, Ahmann GJ, Greipp PR, Jalal SM, Westendorf JJ, Katzmann JA (1993). Coexistence of aneuploid subclones within a myeloma cell line that exhibits clonal immunoglobulin gene rearrangement: clinical implications. Cancer Res.

[22] Namba M, Ohtsuki T, Mori M, Togawa A, Wada H, Sugihara T (1989). Establishment of five human myeloma cell lines. In Vitro Cell Dev Biol.

[23] Matsuoka Y, Moore GE, Yagi Y, Pressman D (1967). Production of free light chains of immunoglobulin by a hematopoietic cell line derived from a patient with multiple myeloma. Proc Soc Exp Biol Med.

[24] Leung-Hagesteijn C, Erdmann N, Cheung G, Keats JJ, Stewart AK, Reece DE (2013). Xbp1s-negative tumor B cells and pre-plasmablasts mediate therapeutic proteasome inhibitor resistance in multiple myeloma. Cancer Cell.

[25] Meister S, Schubert U, Neubert K, Herrmann K, Burger R, Gramatzki M (2007). Extensive immunoglobulin production sensitizes myeloma cells for proteasome inhibition. Cancer Res.

[26] Obeng EA, Carlson LM, Gutman DM, Harrington WJ, Lee KP, Boise LH (2006). Proteasome inhibitors induce a terminal unfolded protein response in multiple myeloma cells. Blood.

[27] Kambhampati S, Wiita AP. Lessons learned from proteasome inhibitors, the paradigm for targeting protein homeostasis in cancer. Adv Exp Med Biol. 2020, in press. 10.1007/978-3-030-40204-4_10 32297217

[28] Chapman MA, Lawrence MS, Keats JJ, Cibulskis K, Sougnez C, Schinzel AC (2011). Initial genome sequencing and analysis of multiple myeloma. Nature.

[29] Lohr JG, Stojanov P, Carter SL, Cruz-Gordillo P, Lawrence MS, Auclair D (2014). Widespread genetic heterogeneity in multiple myeloma: implications for targeted therapy. Cancer Cell.

[30] Walker BA, Mavrommatis K, Wardell CP, Ashby TC, Bauer M, Davies FE (2018). Identification of novel mutational drivers reveals oncogene dependencies in multiple myeloma. Blood.

[31] Rajan AM, Rajkumar SV (2015). Interpretation of cytogenetic results in multiple myeloma for clinical practice. Blood Cancer J.

[32] Zhan F, Huang Y, Colla S, Stewart JP, Hanamura I, Gupta S (2006). The molecular classification of multiple myeloma. Blood.

[33] Billadeau D, Jelinek DF, Shah N, LeBien TW, Van Ness B (1995). Introduction of an activated N-ras oncogene alters the growth characteristics of the interleukin 6-dependent myeloma cell line ANBL6. Cancer Res.

[34] Mitsiades CS, Mitsiades NS, Bronson RT, Chauhan D, Munshi N, Treon SP (2003). Fluorescence imaging of multiple myeloma cells in a clinically relevant SCID/NOD in vivo model: biologic and clinical implications. Cancer Res.

[35] Lam C, Ferguson ID, Mariano MC, Lin Y-HT, Murnane M, Liu H (2018). Repurposing tofacitinib as an anti-myeloma therapeutic to reverse growth-promoting effects of the bone marrow microenvironment. Haematologica.

[36] Broyl A, Hose D, Lokhorst H, de Knegt Y, Peeters J, Jauch A (2010). Gene expression profiling for molecular classification of multiple myeloma in newly diagnosed patients. Blood.

