# Supplementary material for: Evaluating the efficacy of multiple myeloma cell lines as models for patient tumors via transcriptomic correlation analysis
Source: Leukemia. 2020 Mar 2;34(10):2754–65. doi: 10.1038/s41375-020-0785-1 (PMC7483300; doi:10.1038/s41375-020-0785-1)
Supplement: Supplementary file 1 — Supplementary Materials [file 41375_2020_785_MOESM1_ESM.pdf]

Supplementary Materials

Sarin V. et al., "Evaluating the efficacy of multiple myeloma cell lines as models for patient tumors via transcriptomic correlation analysis."

Contents:

Supplementary Methods	p.2
Supplementary Figures S1-S9	p. 6
Supplementary Dataset Legends	p. 15

Supplementary Methods:

Data collection and normalization
The cell line gene expression file (HMCL66_HTSeq_GENE_Counts.txt) was downloaded from the Keats Lab repository (https://www.keatslab.org/data-repository). For patients the gene expression file (MMRF_CoMMpass_IA13a_E74GTF_HtSeq_Gene_Counts.txt gene) was downloaded from the CoMMpass study (https://research.themmrf.org/). Information on how gene expression data for patients was aligned and count based expression estimates calculated can be found in the (MMRF_CoMMpass_IA13_Methods.pdf) file. We used the 48637 Ensembl IDs that were in both the Keats dataset and the CoMMpass dataset for our analysis.

The file (HMCL69_Preliminary_Mutations_Samtools.xlsx) was utilized to determine which cell lines were mutated for mutational subtype analysis as well as for mutational frequency and the file (MMRF_CoMMpass_IA13a_IGV_All_Canonical_Variants.mut) was used to gather patient data on the same. Translocations of cell lines were determined from the (Myeloma_Cell_Line_Characteristics.csv) file downloaded from (https://www.keatslab.org/myeloma-cell-lines/hmcl-characteristics). For patients this data was derived from the (MMRF_CoMMpass_IA13a_Delly_Structural_Calls.txt) file (see below). Patient annotations for progressive disease and Serum M-protein levels came from the (MMRF_CoMMpass_IA13_PER_PATIENT_VISIT.csv) file. Patient annotations for ISS staging came from the (MMRF_CoMMpass_IA13_PER_PATIENT.csv) file.

Data was normalized using vst, the DESeq2 (Love *et al.*, *Genome Biol* (2014) 15:550) wrapper for varianceStabilizingTransformation function in R.

Correlation Analysis
We filtered low count genes by only retaining genes that had greater than 1 counts per million (CPM) in 2 or more samples, leading to analysis of almost exclusively protein-coding genes. We then normalized by variance stabilizing transformation before utilizing the 5000 most variably expressed genes as determined by the interquartile range (IQR) to compare cell lines and newly diagnosed patient samples using Spearman's rank correlation.

We chose the 5000 most variable genes based on previous studies (Yu et al., *Nat Commun* (2019) 10:3574; Chen et al., *BMC Med Genom* (2015) 8:S5). However, we did also analyze the effect of instead choosing the top 10000 most variably expressed genes. In this wider analysis we found the correlation coefficients generally increased but there was an insignificant shift of overall rankings vs. the top 5000 (not shown). For consistency with prior studies in the field we therefore chose to complete our analyses using the 5000 most variable genes.

Subtype Analysis

Translocation and mutation information for cell lines was annotated by the Keats Lab. Mutational information for patients were also found annotated in the CoMMpass dataset. For patient translocation information, we utilized the DELLY VCF files to determine translocations as previously described (Barwick *et al.*, *Nat Commun* (2019) 10:1911). Translocations with homology of 80% or more in any 100bp window within 1 kb of the translocation breakpoint were removed. Average mappability (determined from ENCODE30 20bp mappability tracks) of less than 20% across 1 kb on either side of the translocation breakpoint were removed. Finally, translocations were visually inspected and compared to matched normal tissue and translocations with sequencing anomalies were removed.

ISS staging, serum M protein levels, and progressive disease annotation were also annotated in the CoMMpass database.

For subsets of patients based on ISS staging, serum M-protein levels, and those annotated as having progressive disease in CoMMpass, we compared mean correlation coefficients of patients in each clinical category to all cell lines. For translocation and mutational analysis we correlated cell lines annotated as carrying each genomic aberration, per Keats lab data, to patients annotated as either having or not having the same aberration. The Wilcoxon rank-sum test was used to assess the differences between the groups. Variance was similar between groups.

We also analyzed molecular subtypes previously defined in multiple myeloma patient tumors based on microarray gene expression (Zhan *et al.*, *Blood* (2006) **108**:2020). To classify our samples into the Zhan et al. molecular subtypes, we applied the Nearest Template Prediction method (Hoshida, *PLoS ONE* (2010) 5:e15543.) as implemented by the CMScaller R package. To generate the subtype templates, we used the top 50 over-expressed genes defining each subtype as described in the Supplementary Material for the Zhan et al. manuscript and intersected them with the genes profiled in our cell line and patient samples. We then quantile-normalized, centered and scaled the gene expression data for the patient samples and cell lines separately utilizing the ematAdjust function in the CMScaller package. Finally, we used the ntp function to classify the patient and cell line samples into molecular subtypes. Briefly, this method involves calculating the cosine distance between the sample and each subtype template and then assigning the sample to the subtype with the smallest template distance. Prediction confidence for each sample was estimated from a null-distribution estimated by permutation tests (n=1000).

For the unsupervised hierarchical clustering subtype analysis, we clustered the normalized cell line gene expression profiles using the 5,000 most variable genes as described in the correlation analysis section. We used complete-linkage for the hierarchical clustering method and Euclidian for the distance metric.

CCLE Cross-Check

Correlation analysis was done for overlapping 25 cell lines utilizing the same 5000 genes as determined to be most variably expressed. The cell line gene expression file (CCLE_RNAseq_genes_counts_20180929.gct) was downloaded from the CCLE data

repository (https://portals.broadinstitute.org/ccle/data). We conducted a Spearman's rank correlation for all newly diagnosed patients utilizing the count-based expression estimates as derived from CCLE data and then compared the rankings and mean correlations of the cell lines as analyzed with the original Keats lab data.

Gene Set Enrichment and Gene Ontology Analysis
Differentially expressed genes were determined by the likelihood ratio test method with upper quartile normalization as outlined in the edgeR user's guide. We ranked our genes by log-fold change and conducted our analysis on the 50 hallmark gene sets available on MSigDB (http://software.broadinstitute.org/gsea/msigdb/index.jsp) utilizing the fgsea R package. We considered a gene set to be up or down regulated using log2-fold change > |1| at False Discovery Rate < 0.01. For GO analysis we used the clusterProfiler package and only considered differentially expressed genes with adjusted p-value less than 0.05. We performed an overall analysis for patients and cell lines considering all GO annotations (molecular function, biological process, and cellular component) with redundant GO terms being removed using the simplify function in the clusterProfiler package.

Exome Sequencing Analysis
As annotated in CoMMpass IA13 processed and variant-called exome data, for patients we filtered for all mutations that were annotated as being a missense variant, frameshift variant, stop gained, stop lost, start lost, disruptive in-frame insertions, and deletions so as to narrow our analysis to only deleterious mutations. For cell lines we similarly utilized annotated mutations as annotated in Keatslab processed exome data. Then we filtered for genes which we had information in both cell lines and patients. We present separately the 30 most frequently mutated genes in cell lines as well as genes annotated by Walker et al. (*Blood* (2018) 132:537) as potential driver mutations in myeloma.

Literature results using Google Scholar search
For each cell line in the Keats lab dataset, a Google Scholar (scholar.google.com) search was performed on Oct. 2, 2019 using the term "[cell line] myeloma". The number of unique results returned for this search is reported in Fig. 3D. For cell lines with two versions derived from different tissues from the same patient (for example, KMS-28PE and KMS-28BM) the search only used the common name of the cell line ("KMS28") and the same result number is plotted for both cell line versions.

Cell Culture Conditions
ANBL-6 cells were a kind gift of Dr. Brian Van Ness at the University of Minnesota and originally derived by Dr. Diane Jelinek. Identity was verified by DNA genotyping and confirmed as mycoplasma-free. ANBL-6 were maintained in complete media with RPMI-1640 (RPMI-1640; Gibco) supplemented with 10% fetal bone serum (FBS; Gemini), 1% penicillin-streptomycin University of California San Francisco (UCSF), 2 mM L-Glutamine (UCSF), and 2 ng/mL IL-6 (ProSpec) with 5% CO_2.

Generation of luciferase-labeled ANBL-6

Cell lines stably expressing enhanced firefly luciferase (effLuc) to enable *in vivo* bioluminescence imaging were generated using standard lentivirus transduction methods. Briefly, lentivirus was produced using HEK293T cells transfected with a mixture of transfer plasmid (encoding an effLuc, mCherry, Neomycin-resistance gene expression cassette) and second-generation lentiviral packaging plasmids while cells were ~80% confluent. Transfection of lentivirus plasmids was performed using polyethethylenimine (Transporter-5, Polysciences, 26008-5) at a 4:1, PEI:DNA mass ratio. Virus containing supernatant was harvested after 72-hours and filtered using a 0.45uM syringe filter, then stored at -80°C until used. Transduction of cells was performed by the addition of 1ml of viral supernatant to 1-2E6 cells, supplemented with 8ug/ml polybrene (EMD Millipore, TR-1003-G), then spinfecting at 1000 RCF for 2 hours at 33°C. One day after spinfection the viral supernatant was replaced with fresh media and cells were allowed to recover for several days to allow for transgene expression prior to FACS sorting to achieve a uniform population of effLuc expressing cells. Construct encoding effLuc and mCherry was a kind gift of Dr. Diego Acosta-Alvear, UCSF.

IL-6 titration studies
1e3 ANBL-6 cells were seeded per well in 384 well plates (Corning) using the Multidrop Combi (Thermo Fisher) and incubated for 72 hr with the indicated concentration of IL-6 (ProSpec) in quadruplicate. CellTiterGlo (Promega) was used to measure viable cells per well at 72h and compared to baseline analysis at 0h prior to IL-6 treatment, with plate imaging on the Promega GloMax.

ANBL-6 murine studies
1e6 ANBL-6-luc or MM.1S-luc cells, stably expressing luciferase, were transplanted via tail vein injection into 4 and 6 female NOD.Cg-$Prkdc^{scid}$ $Il2rg^{tm1Wjl}$/SzJ (NSG) mice, respectively. 6-8 weeks old NSG mice were obtained from in-house breeding stocks at UCSF Preclinical Therapeutics Core (PTC) Facility. Tumor burden was assessed through bioluminescent imaging in the UCSF PTC on a Xenogen In Vivo Imaging System (IVIS) at the time points indicated in Fig. 7B. Survival is denoted by the time to development of symptomatic myeloma, at which point sacrifice is required per animal welfare guidelines. All studies were approved by the UCSF Institutional Animal Care and Usage Committee. Sample size chosen based on standard pilot study with no pre-determined statistical model. No randomization or blinding was performed.

Supplementary Figures:

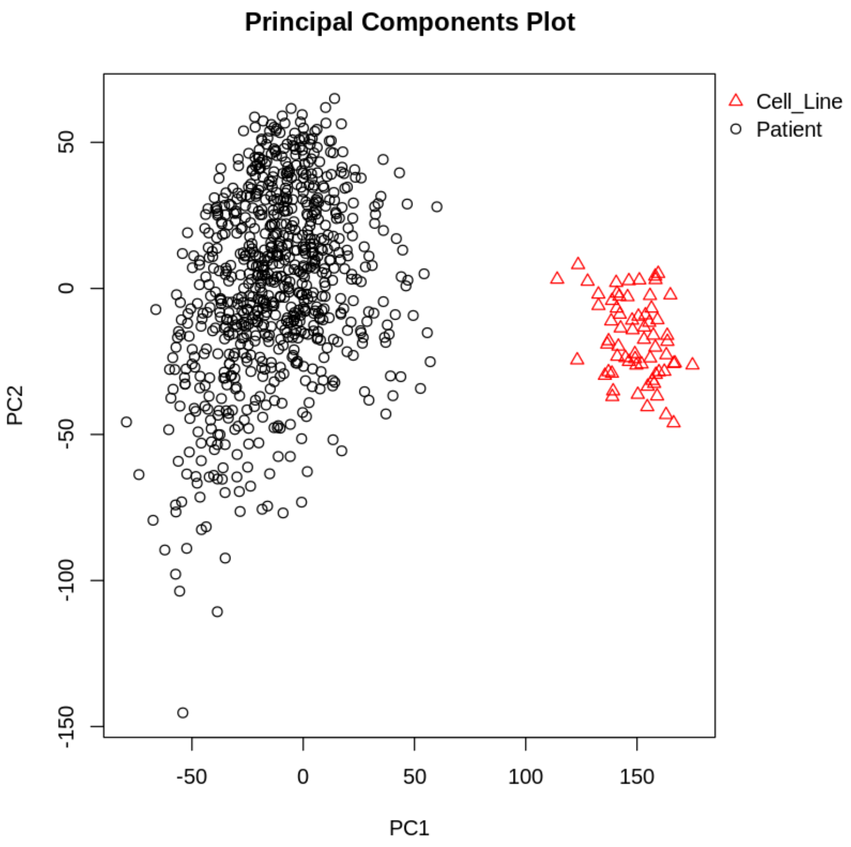

Figure S1. Principal Component Analysis illustrates separation between patient and tumor transcriptomes. Performed based on 5000 most variable genes after variance stabilizing transformation.

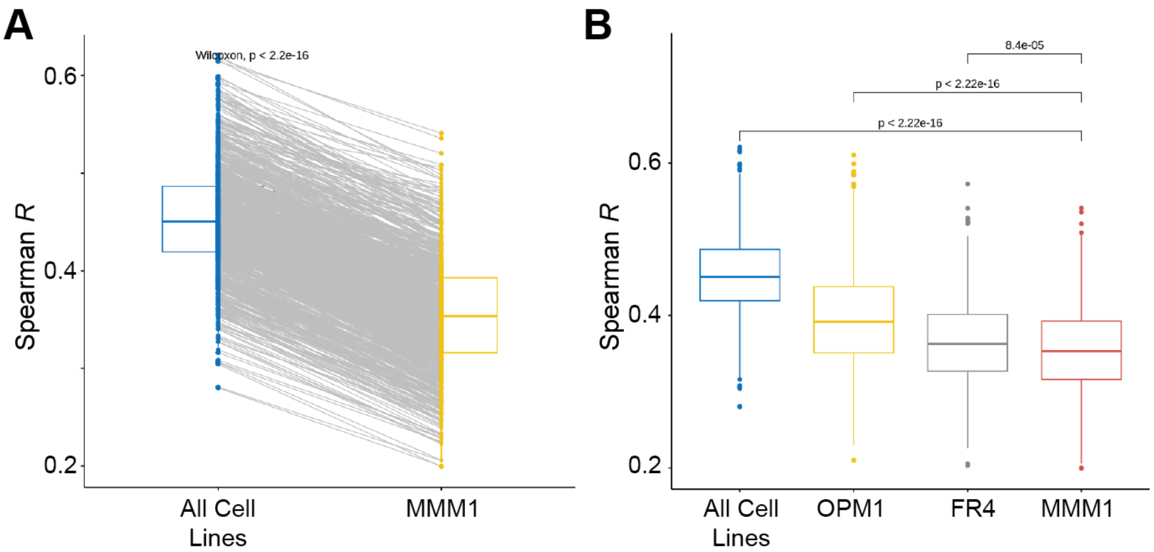

Figure S2. MMM1 is the lowest-ranking cell line. A. Analysis across all patient correlations versus this lowest-ranking line demonstrates significantly decreased patient representation of MMM1. **B.** Comparative analysis of the bottom three cell lines (OPM-1, FR4, MMM1) demonstrates that FR4 and MMM1 are significantly less representative of patient tumor than even OPM1. *p*-values by Wilcoxon test.

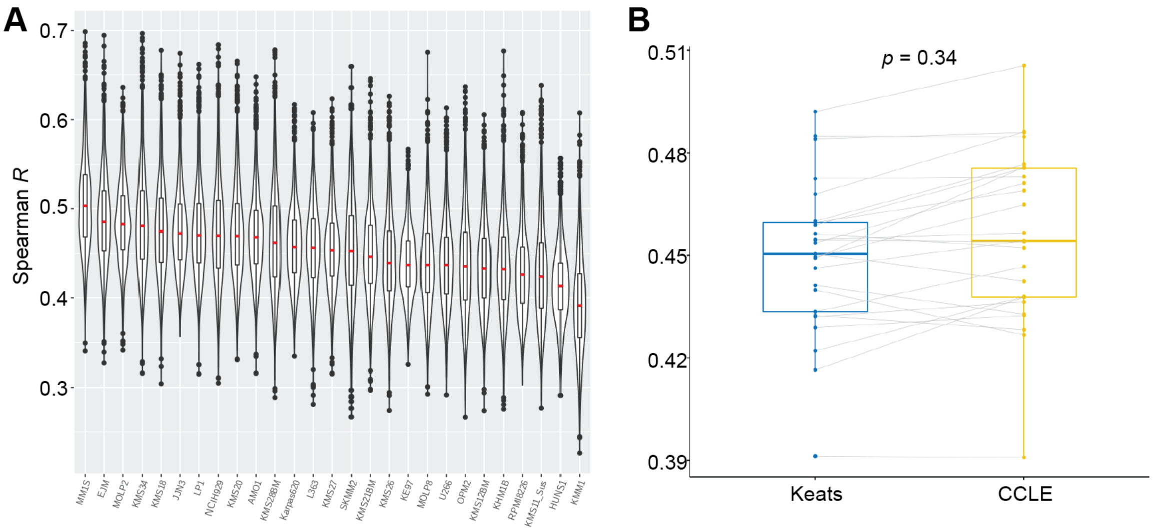

Figure S3. Ranking cell line similarity to patient tumor in CoMMpass using CCLE transcriptome data. A. Similar to Fig. 3A, correlation analysis of the CCLE and CoMMpass transcriptome data. Each sample in the violin plot corresponds to the Spearman correlation between one cell line and one primary tumor sample using the 5000 most variable genes. In the overlaid boxplot, the red center line depicts the median, the box limits depict the upper and lower quartiles, and the whiskers depict 1.5 times the interquartile range. **B.** Comparison of cell line ranking for the 25 lines included in both the CCLE and Keats datasets demonstrates no significant change in median correlation for each line as measured using each cell line dataset (*p*-value by Wilcoxon test).

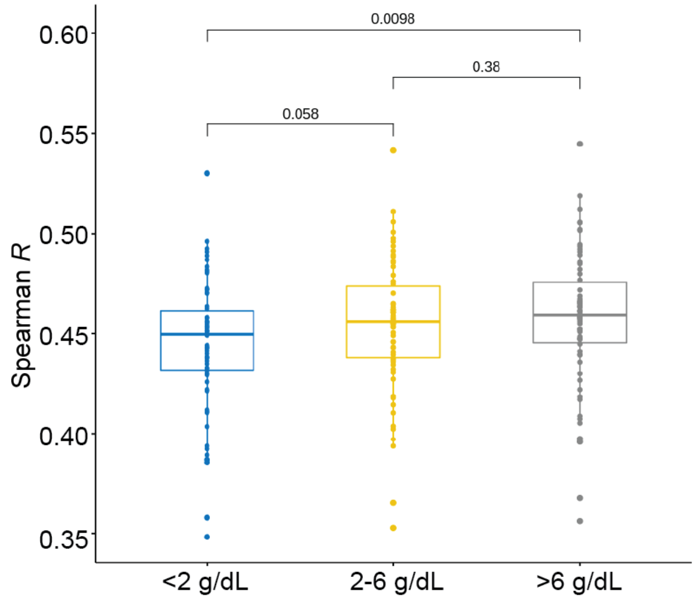

Figure S4. Patient M-spike at diagnosis correlates with increased similarity to cell lines. Box plots indicate median Spearman R for each cell line (dots) versus all patients as annotated in CoMMpass as having the indicated M-spike at diagnosis. Patient tumors associated with M-spike >6 g/dL have significantly greater similarity to cell lines than those with <2 g/dL. *p*-values by Wilcoxon test.

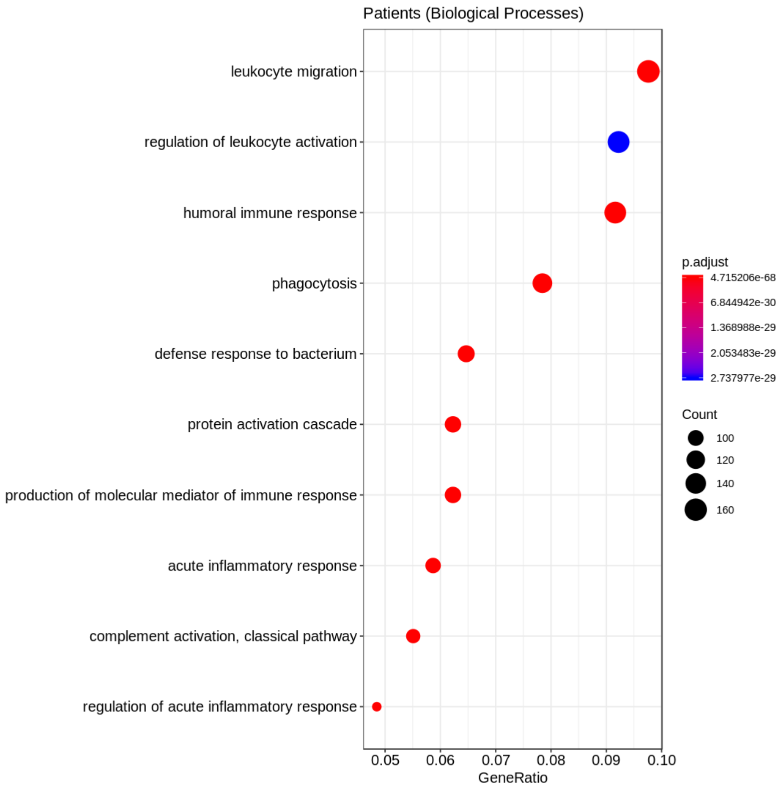

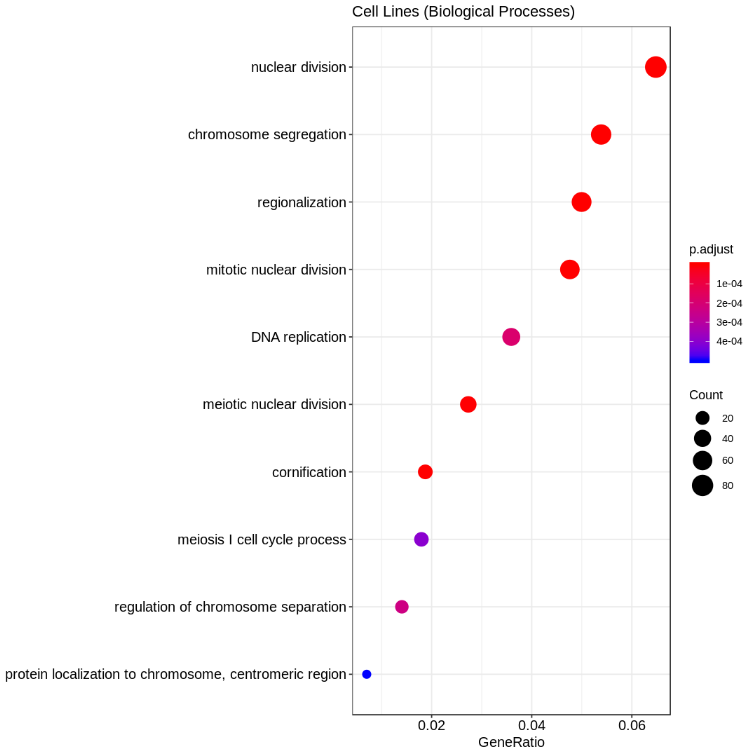

Figure S5. Gene Ontology Analysis recapitulates same upregulated biological functions in patient tumor (above) and cell lines (below) as found by GSEA (Fig. 5D).

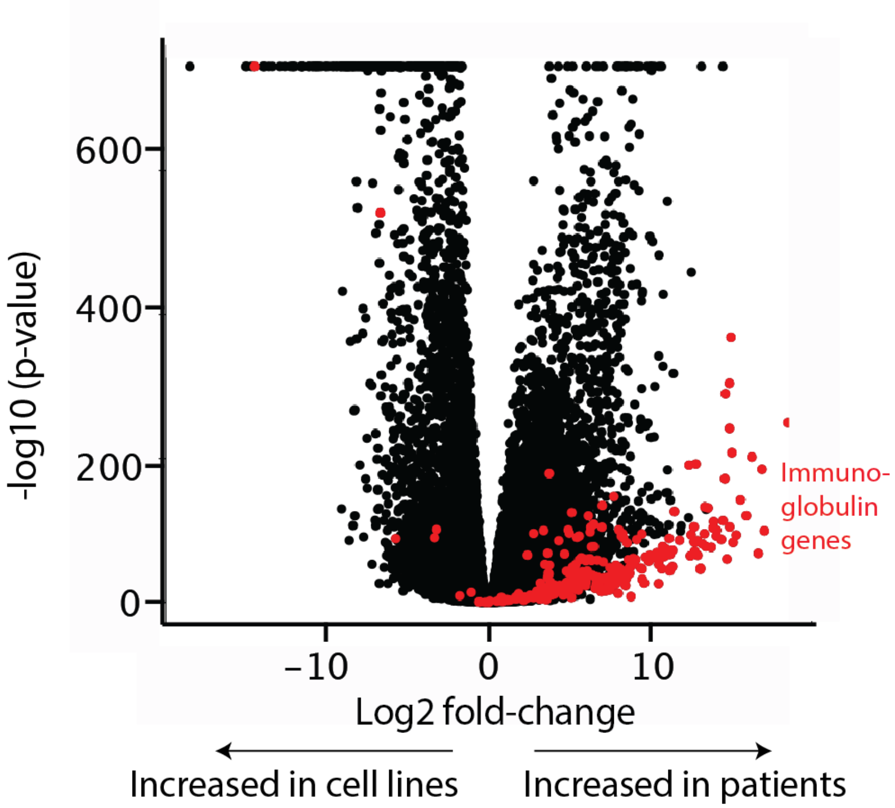

Figure S6. Overall aggregate comparison of gene-level expression between all patients and cell lines. Immunoglobulin genes, encoding different heavy and light chain isoforms (gene names beginning with "IGH", "IGL", and "IGK"), are highly represented among genes with increased expression in patients versus cell lines (also see Dataset S2). Comparison made using EdgeR tool on RNA-seq data.

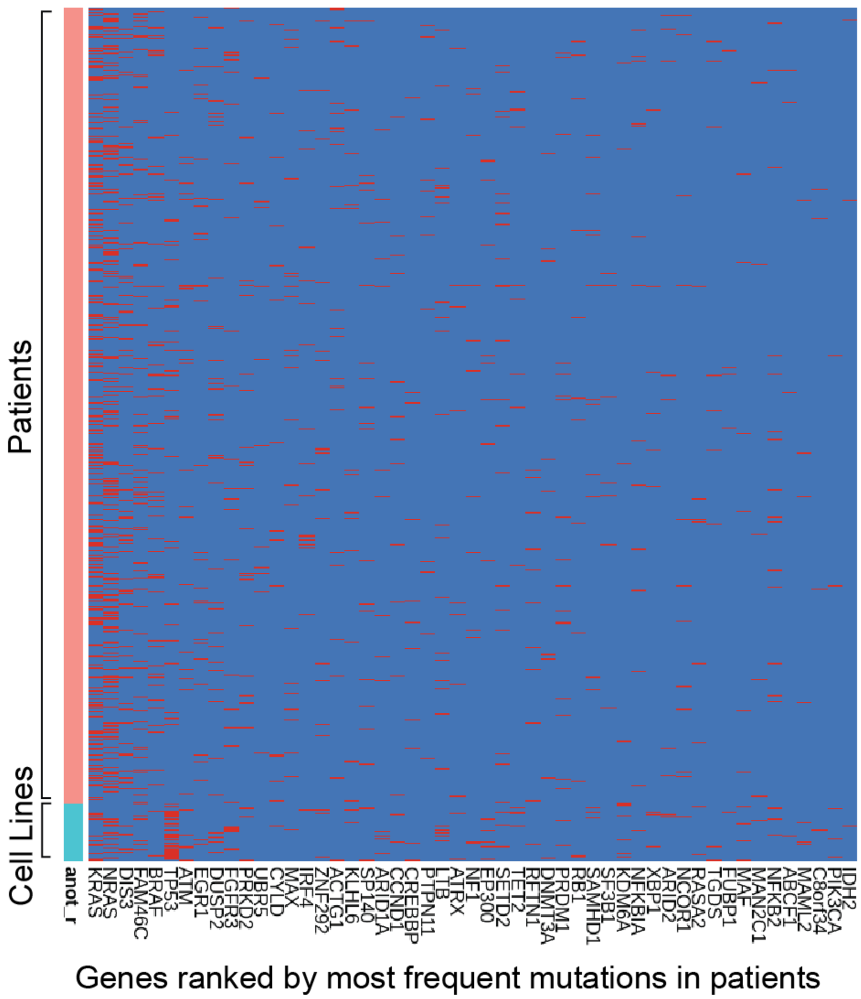

Figure S7. Mutation frequency in both cell lines and patients for most-common mutations detected in patient samples. Patient tumor mutation frequency as characterized by Walker *et al.* (*Blood*, 2018) and ordered by most to least frequent in patient samples. Red = mutation detected compared to hg19 reference genome. Blue = no mutation.

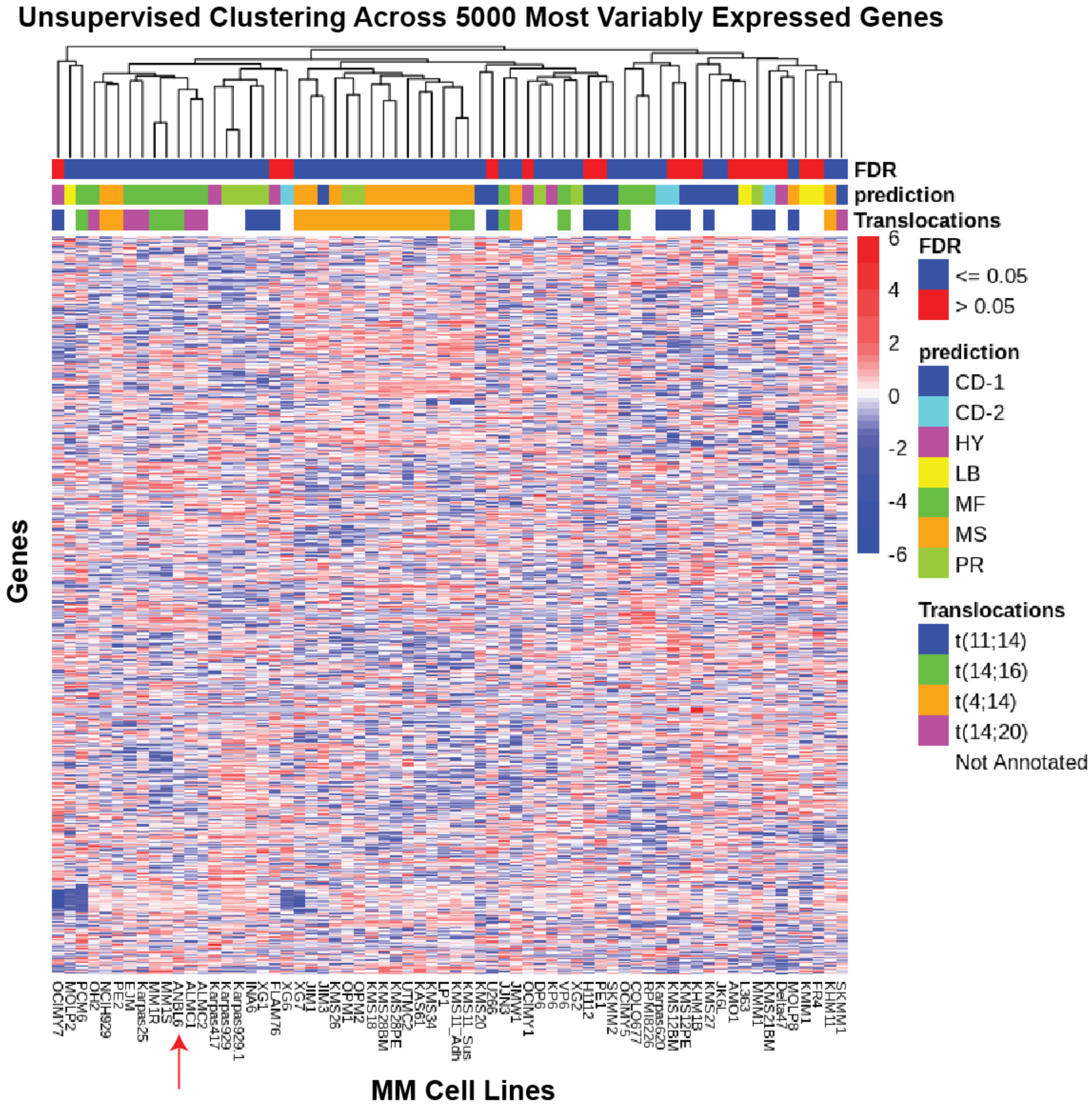

Figure S8. Unsupervised hierarchical clustering of MM cell line transcriptomes. Cluster analysis performed in a similar fashion to that in Fig. 6C, though here the y-axis represents all 5000 genes included for the analysis in Fig. 3A, instead of specific gene lists for each subtype from Zhan et al. In contrast to the patient analysis of Zhan et al., only subtle gene expression signatures appear to distinguish different cell line clusters (x-axis). ANBL-6 noted with red arrow. Heatmap scalebar reflects log2-normalized gene expression data with 0 as the median in each row.

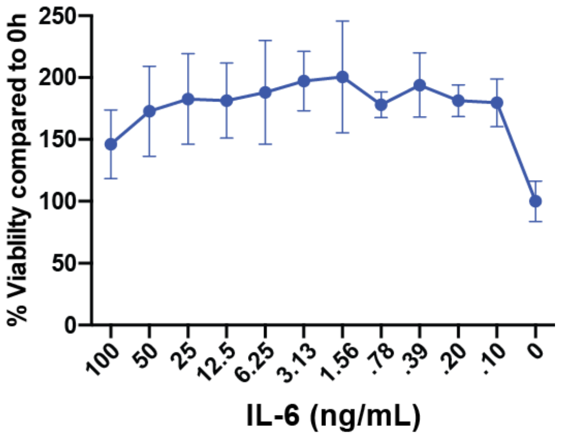

Figure S9. Proliferation of ANBL-6 across IL-6 concentrations. Our results confirm that luciferase-labeled ANBL-6 proliferates *in vitro* at IL-6 concentrations as low as 0.1 ng/mL (n = 4 per concentration; CellTiterGlo measured at 72h), as compared to baseline at 0h. No proliferation is observed without IL-6 (i.e. same number of cells present at 72h as at 0h).

Supplementary Dataset Legends:

Supplementary Dataset 1 (.xlsx file): *Sheet 1:* Data summary for patient-cell line transcriptional Spearman correlations including mean, median, and interquartile ranges. *Sheet 2:* Rankings of citations of each cell line per Google Scholar search. *Sheet 3:* Data summary for patient-cell line transcriptional Spearman correlations versus 25 overlapping myeloma cell lines in CCLE.

Supplementary Dataset 2 (.xlsx file): Differential expression data (log2-fold change and *p*-value) from EdgeR analysis for all genes as compared between cell lines and patient samples, and displayed in Fig. S6.
